# Advanced magnetic resonance neuroimaging techniques: feasibility and applications in long or post-COVID-19 syndrome - a review

**DOI:** 10.1097/MS9.0000000000001808

**Published:** 2024-02-09

**Authors:** Sana Mohammadi, Sadegh Ghaderi

**Affiliations:** a Department of Medical Sciences, School of Medicine, Iran University of Medical Sciences; b Department of Neuroscience and Addiction Studies, School of Advanced Technologies in Medicine, Tehran University of Medical Sciences, Tehran, Iran

**Keywords:** DTI, fMRI, post-COVID-19, PWI

## Abstract

Long-term or post-COVID-19 syndrome (PCS) is a condition that affects people infected with SARS‑CoV‑2, the virus that causes COVID-19. PCS is characterized by a wide range of persistent or new symptoms that last months after the initial infection, such as fatigue, shortness of breath, cognitive dysfunction, and pain. Advanced magnetic resonance (MR) neuroimaging techniques can provide valuable information on the structural and functional changes in the brain associated with PCS as well as potential biomarkers for diagnosis and prognosis. In this review, we discuss the feasibility and applications of various advanced MR neuroimaging techniques in PCS, including perfusion-weighted imaging (PWI), diffusion-weighted imaging (DWI), susceptibility-weighted imaging (SWI), functional MR imaging (fMRI), diffusion tensor imaging (DTI), and tractography. We summarize the current evidence on neuroimaging findings in PCS, the challenges and limitations of these techniques, and the future directions for research and clinical practice. Although still uncertain, advanced MRI techniques show promise for gaining insight into the pathophysiology and guiding the management of COVID-19 syndrome, pending larger validation studies.

## Introduction

Highlights

- Diffusion tensor imaging identifies disrupted white matter integrity.
- Functional magnetic resonance imaging identify altered connectivity.
- Perfusion-weighted imaging identifies reduced cerebral blood volume.
- Neuroimaging biomarkers correlate with the severity of neurological symptoms.

Post-COVID-19 (PCS) is a long-term condition that affects some people who have been infected with coronavirus 2 of the SARS-CoV-2, the virus that causes COVID-19^1–3^. PCS, also known as post-COVID condition (PCC) or long COVID-19, refers to the persistence or emergence of new symptoms 3 months following the initial SARS-CoV-2 infection, and these symptoms persist for a minimum of 2 months without any other apparent cause^4–6^. It is characterized by a wide range of 60 heterogeneous psychological physical symptoms^5,7^ including fatigue^8,9^. These symptoms may either be new-onset following initial recovery or persist from the initial illness, and may fluctuate or relapse over time^10–12^.

PCS can affect multiple organs and systems, including the nervous system, and cause various neurological and neuropsychiatric sequelae^1,13^. The exact pathophysiology of PCS and its neurological manifestations are not fully understood, but it may involve inflammatory, vascular, hypoxic, and neuropsychiatric mechanisms^7,14^. Brain fog is a term used to describe the long-term symptoms of COVID-19, which persist after their initial infection^15,16^. It includes loss of attention, concentration, memory, and mental sharpness, which affect cognitive functions such as memory and mental clarity^15,17^. This can make daily tasks difficult and is a common neurological manifestation of the post-COVID-19 syndrome. Brain fog can be severe enough to impede a patient’s ability to return to their pre-COVID life^16,18^.

In the context of PCS, advanced magnetic resonance imaging (MRI) techniques can provide valuable information on the structural and functional changes in the brain associated with PCS, as well as potential biomarkers for diagnosis and prognosis^19,20^. Advanced MRI techniques can provide qualitative and quantitative MRI biomarkers that reflect the microstructural, functional, and hemodynamic properties of the brain tissue^21,22^. These techniques have been extensively used in various clinical applications, including the diagnosis and treatment of neurological disorders^21^. These advanced neuroimaging modalities can reveal subtle brain alterations that may underlie the cognitive, emotional, sensory, and motor impairments observed in patients^19,23^.

Previous MR neuroimaging studies have focused mainly on the acute stage of COVID-19, while recent studies have begun to investigate brain changes in recovered patients^24^. Advanced MRI techniques can identify widespread white matter (WM) and grey matter (GM) abnormalities in areas crucial for cognitive processing and integration^25^. Difusion-based imaging methods, including diffusion-weighted imaging (DWI), diffusion tensor imaging (DTI), and tractography, can measure WM integrity and connectivity, which are impaired in patients with PCS, indicating axonal damage and fibre disruption^26,27^. Functional MRI (fMRI) can measure brain activity and functional connectivity (FC), which are altered in individual with PCS, indicating functional reorganization and compensation^28^. Perfusion-weighted imaging (PWI) can measure cerebral blood flow (CBF) and cerebral blood volume (CBV), which have been shown to decrease in PCS, indicating impaired vascular supply^20^. Other advanced MRI techniques, such as susceptibility-weighted imaging (SWI) and quantitative susceptibility mapping (QSM), can also provide valuable information on structural changes in brain tissue^29,30^.

### Rationale

Based on our literature search, various controversies have been reported in neuroimaging studies. Yiping *et al*.^31^ reported higher bilateral grey matter volume and no significant changes in WM volume in COVID-positive patients. However, a study by Douaud *et al*.^32^ found significant effects of SARS-CoV-2 infection on brain imaging, including reduced grey matter thickness and global brain size. Notably, a scoping review showed perivascular spaces, microbleeds, and WM lesions in the long-term effects of COVID-19^5^. However, the results are heterogeneous because of the low agreement of MRI abnormalities in PCS.

Thus, in this review article, we discuss the feasibility and applications of various advanced MR neuroimaging techniques in PCS research, including DTI, tractography, fMRI, and PWI. We summarize the current evidence on neuroimaging findings in PCS, the challenges and limitations of these techniques, and the future directions for research and clinical practice. By integrating the results across the full spectrum of advanced MR neuroimaging modalities, we aimed to delineate a comprehensive profile of possible COVID-19-induced brain alterations.

## Advanced MR neuroimaging techniques

### Diffusion-based Imaging and tractography

Diffusion-based imaging methods, such as DWI and DTI, have been employed to examine the diffusion of water molecules in the brain^33^. These methods provide insight into the integrity of WM tracts (WMTs) and their connectivity at the microstructural level^34^. DTI, a subtype of DWI, allows for the reconstruction of WMTs in the brain using tractography^35^.

Several data metrics are commonly used to understand these techniques further.

- Fractional anisotropy (FA): FA measures the degree of directionality of water diffusion within the tissues. This provides information about the microstructural organization of WMTs. High FA values indicate well-organized anisotropic diffusion, whereas low values suggest more isotropic diffusion^33,36^.
- Mean diffusivity (MD): MD quantifies the overall magnitude of water diffusion. It represents the average diffusion rate of water molecules within a region. Elevated MD values can indicate tissue damage or reduced cell density^37^.
- Radial diffusivity (RD): RD measures the water diffusion perpendicular to the WMTs. It can be a sensitive marker for demyelination or axonal injury, as increased RD is associated with these processes^38^.
- Axial diffusivity (AD): AD represents water diffusion parallel to the WMTs. Changes in AD reflect changes in the axonal integrity and growth. Reduced AD may indicate axonal damage^39^.

These data metrics and biomarkers are crucial for interpreting DTI results and understanding microstructural changes in the brain. Recent studies in the context of COVID-19 and post-COVID-19 syndrome have shown that these indices can be employed to monitor changes in brain microstructure over time, offering valuable insights into the neurological effects of the disease^40^.

Tractography utilizes DTI data to visualize the three-dimensional arrangement of WM tracts in the brain^41^. Previous studies have shown that diffusion-based imaging and tractography are useful in several clinical applications such as WMT visualization^33,41,42^. In the context of COVID-19 and post-COVID-19 syndrome, recent studies have revealed that diffusion-based imaging techniques can offer significant insights into the structural changes in the brain^2^. Recent studies have demonstrated that DTI can be employed to monitor changes in the brain microstructure of patients who have recovered from COVID-19 over time^27,43^.

### Functional MR imaging

fMRI is a powerful imaging technique that provides insights into brain function^44^. Measures of changes in blood flow and oxygenation enable researchers to map brain activity^45^. fMRI is widely used in the study of brain disorders, and its applications extend to understanding the effects of diseases such as COVID-19 and post-COVID-19 syndromes^32,46^. fMRI allows researchers to map brain regions associated with specific tasks or stimuli^32^. It can be used to study cognitive impairments and changes in brain activation patterns in patients^46^. Resting-state fMRI (rs-fMRI) is a valuable tool for examining intrinsic brain connectivity. It can help identify alterations in the default mode network that may be affected by PCS^47^. Rs-fMRI metrics, such as the amplitude of low-frequency fluctuation (ALFF) and fractional ALFF (fALFF), are widely used to detect brain abnormalities based on signal fluctuations^48^. The ALFF is an rs-fMRI indicator used to measure the regional intensity of spontaneous fluctuations in the BOLD signal. This technique helps identify the spontaneous neural activity of specific brain regions and physiological states^49,50^.

Furthermore, fMRI is essential for tracking changes in brain activity and connectivity over time. This is particularly relevant in understanding the long-term neurological effects of COVID-19^51^.

fMRI can be used to explore changes in FC between brain regions, shedding light on the neurological consequences of the virus. FC is a metric that measures the temporal correlation between different brain regions^51,52^. In summary, fMRI is a crucial tool for the investigation of brain function and its adaptations in response to COVID-19. This can provide valuable data for the study of PCS, offering a deeper understanding of the neurological implications of the disease. Rs-fMRI metrics such as ALFF and fALFF, as well as FC are some of the metrics that can be used to detect abnormalities and changes in intrinsic connectivity^23,47,51–58^.

### Perfusion-weighted imaging

PWI is a vital neuroimaging method that evaluates CBF in the brain^20^. It provides critical information on the state of perfusion of the brain, making it a valuable tool for understanding vascular changes and their implications in various neurological conditions, including post-COVID-19 syndrome^20,59–61^. PWI encompasses several techniques including dynamic contrast-enhanced (DCE) imaging, dynamic susceptibility contrast (DSC) imaging, and arterial spin labelling (ASL)^62^. PWI, which includes DCE, DSC, and especially ASL, is an indispensable set of neuroimaging techniques for examining cerebrovascular changes in post-COVID-19 syndrome. These methods offer a unique window into the perfusion dynamics, shedding light on the vascular aspects of this condition^20,59–61^.

## Neuroimaging findings in PCS

### Structural changes in the brain associated with PCS

Diffusion MRI studies have found mixed results in PCSs compared with healthy controls (HCs). One study showed that increased MD and extracellular free water in WM regions, equivalent to 6–7 years of age, correlated with cognitive impairment^63^. Other studies have found a decrease in MD in the GM and WM regions^23,64^. Studies have reported altered diffusion metrics in PCSs in specific regions and tracts: increased MD and RD in WM tracts related to olfactory dysfunction^65^, increased MD in WM with headaches^64^, and decreased FA in the corpus callosum and WM tracts upon admission to the ICU and headaches^64,66^. Changes found in structural networks and connections: changes in left amygdala FA after hyperbaric oxygen therapy^53^; reduced fibre volume, length, and FA correlated with severity^60,67^. Mixed results were obtained for other diffusion biomarkers: an increase in the volume fraction of the isotropic diffusion compartment^68^, a decrease in apparent fibre density^27^, and a decrease in intracellular water fraction^67^.

In line with these studies, a recent systematic review^69^ suggested a correlation between structural abnormalities, such as WM hyperintensities and cortical atrophy, and memory impairment in various brain regions in COVID-19 survivors. Overall, diffusion MRI showed alterations in WM microstructure and connectivity in PCSs compared to HCs, with changes often correlating with symptoms such as cognitive impairment. However, the causal relationship remains unclear, and further longitudinal investigations, particularly controlled studies combined with correlational analyses, are needed to provide additional evidence.

### Functional changes in the brain associated with PCS

Studies have found altered ALFF in PCSs with cognitive impairment compared to HCs, including increased ALFF in the occipital and temporal regions and decreased ALFF in the frontal regions^51^. Abnormal ALFF affects the FC between regions^51^. Research has shown that reduced connectivity between the parahippocampal gyri and frontal cortex in PCSs is correlated with memory impairment^23^. Another study found differences in resting-state FC between cognitive complainers and non-complainers^56^. One study showed decreased connectivity between networks, such as the default mode, frontoparietal, and somatomotor networks, in PCSs after hyperbaric oxygen therapy^53^. Connectivity changes are correlated with symptoms and cognition^53^. PCSs with severe symptoms have reduced connectivity between attention, sensory, motor, salience, and default mode networks compared to those with mild symptoms^57^. Patients with moderate symptoms showed increased subcortical-cortical connectivity. Studies have revealed connectivity changes related to olfactory dysfunction in PCSs, including the olfactory network, thalamus, putamen, and piriform cortex^47,55^. Task-based fMRI showed altered activation in the PCSs during working memory tasks, suggesting compensatory recruitment of brain regions^58^. Hospitalization and symptoms are correlated with differences in activation^23,58^. In summary, PCSs shows extensive functional network connectivity changes that correlate with persistent symptoms, such as cognitive impairment and olfactory loss.

### Perfusion findings

All studies use ASL to measure CBF^20,59–61^. Studies have shown reduced CBF in certain brain regions of PCSs compared with HCs, including the thalamus, orbitofrontal cortex, basal ganglia, and frontal, parietal, and temporal cortical regions^20,60,61^. The right hemisphere appeared to be more affected than the left^20^. Individuals with PCS with severe symptoms have decreased CBF in the superior medial frontal gyrus and insula^60^. PCS with olfactory dysfunction has reduced perfusion in the orbital and medial frontal regions associated with the olfactory system^59^. Persistent olfactory dysfunction is associated with decreased perfusion in regions of the olfactory system^59^. PCS with fatigue shows increased CBF in the superior occipital and parietal regions, but a decrease in CBF in the inferior occipital regions^61^. More severe symptoms were correlated with greater hypoperfusion in the frontal areas^20,60,61^. In summary, PCSs demonstrate reduced CBF compared with HCs, particularly in regions related to the frontal and olfactory systems.

## Challenges and limitations

Although advanced neuroimaging techniques show promise for understanding brain changes in individuals with PCS, current research has several challenges and limitations. A primary limitation of DTI, tractography, fMRI, and ASL studies is their small sample size, which restricts the conclusions that can be drawn. To date, most studies have included less than 50 patients. Larger cohorts are required to improve the statistical power and better characterize the heterogeneity of neurological sequelae. Diffusion MRI studies demonstrate inconsistencies in the findings, with reports of increased, decreased, or mixed changes in metrics, such as FA, MD, AD, and RD, across WMTs. This variability may stem from differences in patient populations, severity of the initial COVID-19 illness, timing of scans post-infection, and analysis methods. Standardization of protocols across research centres is needed. Tractography holds promise for mapping specific WM pathway damage in individuals with PCS. However, technical factors can affect tractography results, including the region of interest (ROI) placement, algorithm parameters, and scan acquisition details. Multicenter studies with harmonized tractography protocols are warranted. Although fMRI reveals FC alterations in the brain after COVID-19, the cross-sectional nature of most studies makes it difficult to determine causality. Task-based fMRI findings remain preliminary, and longitudinal fMRI studies are required to clarify the primary pathology versus compensatory changes. Finally, combining advanced MRI modalities and correlating imaging findings with detailed clinical data remains an important priority in the field.

## Future directions for research and clinical practice

The advanced neuroimaging studies reviewed highlight the need for larger longitudinal investigations to clarify the primary pathological brain changes versus compensatory mechanisms in patients with PCS. Large multicenter studies with standardized protocols are critical for establishing consistent imaging biomarkers. Combined tractography-fMRI studies can associate structural disconnections with network dysfunction. Prospective rs-fMRI research is needed to clarify the evolution of these changes over time. Multimodal fMRI and PWI could better elucidate structure-function relationships.

ASL has emerged as a valuable technique in PCS studies, offering a deeper understanding of CBF alterations in the affected regions. ASL, as a non-invasive perfusion imaging method, plays a crucial role in assessing perfusion deficits in PCS^20,59^. This approach enables researchers to identify areas of the brain where CBF abnormalities may contribute to post-COVID-19 neurological symptoms. ASL’s advantage lies in its ability to provide quantitative measurements of CBF, making it a valuable tool for identifying areas of decreased perfusion and understanding their clinical implications. Integrating ASL into comprehensive neuroimaging studies alongside DTI and fMRI offers a holistic approach to investigate the structural and functional changes in post-COVID-19 brains, ultimately advancing our understanding of the neurological effects of the virus.

In addition, SWI is an MRI technique that is sensitive to iron deposition, blood products, and calcium, making it valuable for detecting microhemorrhages and pathological mineral accumulations^34^. SWI magnitude and phase data can be used to generate QSM that quantify the magnetic susceptibility of tissues^70–73^. Initial studies revealed SWI microhemorrhages in COVID-19 patients, indicating microvascular damage^29^. Moving forward, SWI and QSM should be utilized to evaluate cerebral microbleeds, iron deposition, and calcium accumulation to better understand potential cerebrovascular pathology and neuroinflammatory-neurodegenerative processes in brains affected by COVID-19.

Clinically, advanced neuroimaging could aid in diagnosis, prognostication, and treatment monitoring if imaging biomarkers are validated. The severity of changes in patients could help identify their risk levels. By repeatedly scanning their brains, rehabilitation efforts can be directed toward the affected networks. Neuroimaging is crucial for monitoring further development beyond the initial post-COVID phase. In general, advanced MRI techniques that combine different parameters, such as DTI, fMRI, PWI, and tractography, have great potential for revealing the intricate neurological effects of COVID-19 on the brain.

## Conclusions

Current research has demonstrated emerging alterations in brain structure, function, and perfusion in PCS compared with healthy individuals. Larger longitudinal studies applying standardized, multimodal imaging protocols are still needed to validate biomarkers and clarify the evolution of pathology over time. Integrating advanced MRI with clinical data may help determine the causes of persistent neurological symptoms. As evidence accrues from well-powered investigations, neuroimaging has the potential to aid in the diagnosis, prognosis, and monitoring of recovery. Collaborative research utilizing cutting-edge neuroimaging globally could substantially further our understanding of heterogeneous brain alterations in the emerging long-term condition of post-COVID-19 syndrome.

## Ethical approval

Ethics approval was not required for this review.

## Consent

Informed consent was not required for this review.

## Source of funding

Not applicable.

## Author contribution

Conceptualization: S.G.; Methodology: S.Gh.; Software: S.G., S.M.; Validation: S.G., S.M.; Formal analysis: S.G.; Investigation: S.G., S.M.; Resources: S.G., S.M.; Data curation: S.G., S.M.; Writing—original draft: S.G., S.M.; Writing—review and editing: S.G., S.M.; Visualization: S.G.; Supervision: S.G.; Project administration: S.G., S.M.

## Conflicts of interest disclosure

None of the authors have any conflict of interest to disclose.

## Research registration unique identifying number (UIN)

Not applicable.

## Guarantor

Sadegh Ghaderi.

## Data availability statement

This article contains all of the data produced or analyzed during this investigation. Any further inquiries should be forwarded to the corresponding author.

## Provenance and peer review

Not commissioned, externally peer-reviewed.
